# Solubility, structural properties, and immunomodulatory activities of rice dreg protein modified with sodium alginate under microwave heating

**DOI:** 10.1002/fsn3.1105

**Published:** 2019-07-15

**Authors:** Xiangyong Meng, Tingting Li, Teng Song, Chang Chen, Chandrasekar Venkitasamy, Zhongli Pan, Huien Zhang

**Affiliations:** ^1^ College of Environmental Science and Engineering Anhui Normal University Wuhu China; ^2^ Department of Biological and Agricultural Engineering University of California Davis Davis, CA; ^3^ College of Light Industry and Food Engineering Nanjing Forestry University Nanjing China; ^4^ College of Biological and Environment Science Zhejiang Wanli University Ningbo China

**Keywords:** immunomodulation, maillard reaction, rice dreg protein, solubility, structure

## Abstract

This research aims to investigate the solubility, structural properties, and immunomodulatory of rice dreg protein (RDP) modified with sodium alginate. The modification was done by wet heating assisted with microwave treatment. The solubility, emulsifying properties at pH 2–12, amino acid composition, molecular weight distribution, circular dichroism (CD) spectroscopy, and FTIR spectra of modified RDP were analyzed and discussed. Results showed that Maillard reaction could significantly enhance the solubility and emulsifying capacity of RDP. Further, an animal model for cyclophosphamide‐induced immunodeficiency was designed to evaluate the immunomodulatory effect of modified RDP. It is therefore suggested that modified RDP could improve the immunomodulatory effect of immunosuppressed mice, and the immunomodulation was concentration dependent, being generally enhanced by increased concentrations. This research revealed that glycosylation modification of RDP through Maillard reaction by wet heating assisted with microwave treatment may be successfully applied to improve the physicochemical properties and bioactive benefits of the final product.

## 1 INTRODUCTION

Rice protein is a high‐quality protein origin due to its unique nutritional value and hypoallergenic properties when compared with other grain and legume proteins (Fiocchi et al., 2003; Zhang, Wang, Liu, Gong, & Sun, 2016), which make rice protein suitable as a priority alternative to soy proteins in food processing. Rice dreg, the main by‐product of the production of starch syrup, is a valuable source of protein. The protein content of rice dregs ranges from 50% to 70% under different process conditions. However, RDP always suffers from denaturation caused by high temperature during the liquefaction process, which adversely affects its solubility characteristics. The solubility directly is related to other techno‐properties of proteins, such as gelling properties, emulsifying, and foaming. Therefore, solubility plays a crucial role in developing new technical formulation and functional food.

Structural modifications of proteins by physical methods (i.e., ultrasound, high pressure, and pulsed electric field.), chemical methods (i.e., acylation, glycosylation, and covalent cross‐linking effect.), and enzyme methods have been investigated to improve the technical properties of proteins in the food matrix. Li et al. (2016) investigated the effects of combined treatment of ultrasound and alkaline pretreatments on the structural characteristics of rice protein. The results showed that ultrasound decreased a‐helices but increased β‐sheets and the content of nonhydrophilic amino acids. Zhao et al. (2012) investigated the effects of enzyme type on the size and amino acids of RDP. They reported that the combined treatment with alkaline protease and neutrase enzymes increased the solubility of RDP, but also increased the content of bitter amino acids such as arginine and leucine. Although these technologies successfully improved the solubility of RDP, some undesirable substances, such as bitter hydrolysates produced by chemical or biochemical modifications, could lead to commercial value loss.

Maillard reaction is a nonenzymatic reaction of reducing sugars and available amino acids of proteins without additional extraneous chemicals and has been proven to be very important in producing flavor, aroma, and functional foods. The two types of Maillard reactions are caused by dry heating and wet heating. Dry heating takes several days to form protein–polysaccharide conjugates, making it slower than wet heating. Moreover, the products have a dark brown color, which is not desirable for many functional foods, and the reaction process of dry heating is difficult to control. Therefore, improved RDP modification technologies should be developed for functional foods. Sodium alginate, an important cell wall component isolated from brown marine algae (*Phaeophyceae*), is composed of polymeric sequences of (1–4) linked β‐D‐mannuronate (M) and α‐L‐guluronate (G) residues and has a high affinity for cationic protein molecules (Bokkhim, Bansal, Grøndahl, & Bhandari, 2015; Fioramonti, Perez, Aríngoli, Rubiolo, & Santiago, 2014). Sodium alginate has been extensively used to improve water holding, gelling, and emulsifying properties of food proteins based on the interactions between protein and polysaccharide (Yao et al., 2018).

Usually, plant peptides contain 2–20 amino acid residues and may show a wide range of biological activities such as antioxidant, antihypertensive, and immunoregulation. (Ferri et al., 2017; Shen et al., 2016). In the reported studies, the constituents of rice or by‐products of rice milling such as proteins and peptides have been studied to a certain health‐related bioactivity such as antityrosinase, anti‐inflammatory, antihypertensive, and antioxidant properties (Zhang et al., 2016). Up to now, there are no related reports that rice protein–polysaccharide conjugates with antioxidants could exhibit immunoregulatory activities.

Therefore, the objective of this study was to investigate the effects of glycosylation modification of RDP with sodium alginate through Maillard reaction by wet heating in addition to microwave heating. The effects of glycosylation on the solubility, emulsifying properties, structure, and immunomodulatory activity of modified RDP were investigated. The findings of this research would be beneficial for developing novel techno‐functional additives as added‐value food ingredients.

## 2 MATERIALS AND METHODS

### 2.1 Preparation of RDP

According to the methods of Song and Zhang (2017), RDP was prepared with the following steps. Firstly, the rice dregs were pretreated with 650 U/g lipase (enzymatic activity 10,000 U/g, Jiangsu Ruiyang Group Co., Wuxi, China) at 50ºC with pH 9.0 and hydrolysis time 90 min. Secondly, to improve the purity of protein, the effects of freeze‐thawing pretreatment, enzymatic hydrolysis, ultrasound, and their combinations on the content of protein were investigated according to the methods of Song and Zhang (2017).

### 2.2 Preparation of products with Maillard reaction

RDP was weighed and dispersed in 2% (w/w) deionized water. The pH of the solution was adjusted to 12.0, magnetically stirred for 30 min at 50ºC, then cooled down to room temperature. Sodium alginate was added into the dispersion at a rate of RDP 1.88:1 (w/w), and the pH was reduced to 10.2 with 1 mol/L HCL or 1 mol/L NaOH, magnetically stirred for 10 min. Subsequently, 50 ml of a mixed solution of RDP and sodium alginate was transferred to a 100‐ml flask and placed in an experimental microwave oven with a reflux device. The mixed solution was treated by intermittent microwave heating by the methods reported in our previous paper (Meng, Zhang, Song, & Zhang 2018). At the end of the reaction, the flask was immediately cooled down in an ice bath for 5 min.

### 2.3 Solubility measurement

The solubility of RDP modified with sodium alginate through the heat treatment was determined following the reports of Sun, Yu, Zeng, Yang, and Jia (2011). products with Maillard reaction (PMR) was dispersed in deionized water (1 mg/ml), and then, the pH was adjusted to 2, 4, 6, 8, 10, and 12 using 2 mol/L solutions of HCl and NaOH. Then, the solution was magnetically stirred for 30 min at room temperature and centrifuged for 15 min at 4,500 r/min. The absorbance of the supernatants at 280 nm was determined to reflect the protein content, and the solubility of protein in the supernatants was calculated as a percentage of the total protein content.

### 2.4 Emulsifying stability measurement

As the methods described by Li et al. (2013), the emulsifying stability of PMR was determined with slight modifications. The PMR sample was diluted with pH 8.0 0.05 mol/L PBS to 0.4% protein concentration. Briefly, 9 ml of the sample solution was mixed with 3 ml of soy oil and kept continuous agitation. Then, the coarse emulsion was homogenized for 5 min at 10, 000 g using with a homogenizer to achieve a good emulsion level. The emulsion was, respectively, sampled at 0 and 10 min, and the dilution solution was immediately prepared by adding 5 ml of 0.1% sodium dodecyl sulfate (SDS). Once the emulsion formed (0 min), the absorbance at 500 nm of the diluted emulsion was immediately determined and measured. Then, the emulsion stability (ES) was calculated as follows:

(1) $$\text{ES}=\frac{A_{0}\times 10}{A_{0}-A_{10}}$$

where *A*_0_ is the absorbance of the emulsion (0 min), and A_10_ is the absorbance of the emulsion (10 min).

### 2.5 Amino acid analysis

The methods reported by Li et al. (2016) with slight modifications were used to determine the amino acid composition of PMR. Firstly, 6 mol/L HCl was used to hydrolyze the samples at 110ºC. Then, the hydrolysate was filtered and set to 50 ml with deionized water. The determination of the amino acid content was carried out by the methods described by Li et al. (2013).

### 2.6 Molecular weight distribution

The molecular weight distribution of PMR was analyzed with high‐performance gel permeation chromatography (HPGPC) with a TSK Gel G4000 PWXL column was used to analyze (Li et al., 2013). Before injection, the supernatant was collected and filtered (0.2‐mm Whatman filter) after centrifuging for 10 min at 1,000 g. The mobile phase contained PBS buffer (0.05 mol/L, pH 8.5) with a flow rate of 1 ml/min at 20ºC. Detection was determined at a wavelength of 230 nm.

### 2.7 Circular dichroism spectrum

Briefly, the samples were mixed with PBS buffer (10 mmol/L, pH 7.2) to prepare 0.1 mg/ml solution, and then, the solution was centrifuged for 10 min at 10,000 r/min. A quartz cuvette (1 mm optical path length) was supplemented with nitrogen flux at room temperature (25 ± 1ºC), and the data of circular dichroism (CD) spectra were acquired from 190 to 250 nm. The average spectra data of three scans with PBS (0.01 mol/L, pH 8.0) were used as blank.

### 2.8 FTIR spectra measurement

The FTRI spectra were determined using a Thermo Nicolet iS 50 FTIR spectrometer with full‐band scanning (4,000–400 cm^−1^) according to the method of Ji et al. (2018). One milligram of the samples was mixed with 150 mg of KBr and ground gently with an agate pestle and mortar under an infrared lamp. Next, the sample powder was pressed into a 13‐mm‐diameter disk by applying 15 tons of pressure for 30 s. Then, the spectrum intensities of the secondary structure of PMR were analyzed using Omnic software (version 8.0, Thermo Nicolet Inc., Waltham, MA, USA).

### 2.9 Immune activities of products with Maillard reaction

#### 2.9.1 Animal experiments

Healthy male BALB/c mice (18–20 g) after eight weeks of the birth were purchased from Laboratory Animal Center which is located at Soochow University, then transferred to Jiangnan University. All animal experiments were permitted by the Ethics Committee of Jiangnan University. According to the methods described by Shen, Mao, Chen, Meng, and Ji (2015), mice were randomized to five groups, each with 10 mice: blank group, model group, low‐dose PMR of 80 mg kg^−1^ day^−1^, mid‐dose PMR of 160 mg kg^−1^ day^−1^, and high‐dose PMR of 320 mg kg^−1^ d^−1^. After fed adaptively for 4 days, the blank group of healthy mice were given physiological saline solution once daily for 14 consecutive days. In the first 3 days, all other mice were intraperitoneally injected 100 mg kg^−1^ d^−1^ cyclophosphamide (CY). Then, the model mice were gavaged with physiological saline solution once daily from days 4 to 14. Other three groups of mice, respectively, were intraperitoneally injected with PMR at dose of 80, 160, and 320 mg/kg body weight.

#### 2.9.2 Immune organ indexes

After 24 hr of the last drug administration, the mice were decapitated, and then, the spleen and thymus were immediately weighed after excision from the mice. The thymus and spleen indices were calculated by the following equation:

(2) $${\text{Index (mg g}}^{-1}\text{)}=\frac{\text{Weight of thymus or spleen}}{\text{Body weight}}$$

#### 2.9.3 Lymphocyte proliferation assay

The spleens were removed from the mice, washed with 0.1 mol/L PBS buffer solution (4ºC), and gently grated and filtered with a 40‐μm nylon cell strainer to obtain single‐cell suspensions. Then, the single‐cell suspensions were treated with the lymphocyte separation medium to separate erythrocytes and lymphocytes. Lymphocytes were washed with PBS and resuspended to a final density of 1 × 10^6^ cells/mL in RPMI 1640 medium supplemented with newborn bovine serum (10%), 1% glutamine (200 mmol/L), penicillin (100 U/μL), streptomycin (100 μg/L), and 2‐mercaptoethanol (5 × 10^−5^ mol/L). In a 96‐well plate containing concanavalin A (5 μg/ml), the spleen cells were cultured for 72 hr in 5% CO_2_ atmosphere at 37ºC and further incubated for 4 hr in MTT (3‐[4,5‐dimethylthiazol‐2‐yl]‐2,5 diphenyl tetrazolium bromide, 5 g/L). Afterward, the plate was centrifuged for 15 min at 200×*g* to remove the supernatant. Subsequently, the plate was shaken after the addition of 150 μl dimethyl sulfoxide until all crystals dissolved. The absorbance of samples was measured at 570 nm.

#### 2.9.4 Phagocytic index

According to the methods of Shen et al. (2015), after intravenous injection with diluted India ink (100 ml/kg), the blood samples from the retinal venous plexuses were collected at 2 min and 10 min, respectively. Then, 20 µl of the blood samples from each mouse was added into 2 ml 0.1 Na_2_CO_3_. Subsequently, the absorbance of the mixed solution was measured at 600 nm with 0.1% Na_2_CO_3_ as the blank. The liver and the spleen were weighed, and the phagocytic index was calculated as follows:

(3) $$k=\frac{\log\text{OD1}-\log\text{OD2}}{t_{2}-t_{1}}$$

(4) $$\text{Phagocytic index}\;\alpha =\frac{\text{Body weight}}{\text{Liver weight}-\text{Spleen weight}}\times \sqrt[{3}]{k}$$

### 2.10 Statistical analysis

All analysis was made by one‐way analysis of variance (ANOVA) test using SPSS 22.0. The animal experiment results were expressed as the mean ± *SD* (*n* = 10), and other experimental results were expressed as the mean ± *SD* (*n* = 3). Significant differences were considered statistically at the level of 0.05.

## 3 RESULTS AND DISCUSSION

### 3.1 Solubility of PMR

**Figure 1:**
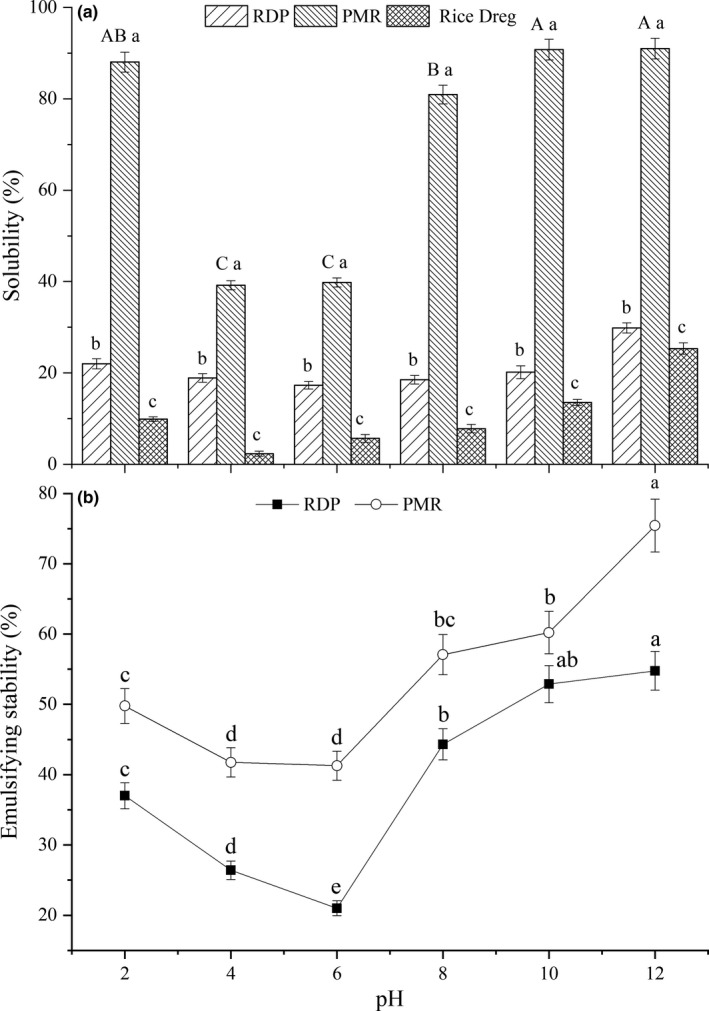
Solubility of rice dreg, RDP, and PMR and emulsifying stability at different pH. ^a–c^The different lowercase superscript letters in (a) mean significant differences between RDP, PMR, and rice dreg (p < 0.05); ^A–C^the different lowercase superscript letters in bars mean significant differences at different pH value (p < 0.05). ^a–e^The different lowercase superscript letters in (b) mean significant differences at different pH vlue (*p* < 0.05)

The solubility of rice dreg, RDP, and PMR at different pH is presented in Figure 1a. The solubility was increased further (*p < *0.05) by the modifications of sodium alginate under microwave radiation when compared to the unmodified RDP and rice dreg. Under the same Maillard reaction conditions, PMR at pH greater than 10 showed the highest solubility. Similarly, PMR at pH 2 showed higher solubility than that of samples with pH 4–6. In addition, the solubility of PMR increased from 39.78% to 90.97% between pH 6 and pH 12, but it decreased from 88.03% to 39.19% between pH 2 and pH 4. It has been reported that the solubility of PMR increased to a higher level than that of the mixture of protein and polysaccharides untreated by means of heat or protein at the same pH (de Oliveira, Coimbra, de Oliveira, Zuniga, & Rojas, 2016). Du et al. (2013) and Mishra, Mann, and Joshi (2001) proposed that the hydration of protein with the conjugation of hydrophilic polysaccharide was improved at the beginning of the Maillard reaction, resulting in improved solubility. That is probably due to the covalent bonding reaction between the amino acid residues of protein and the terminal aldehyde groups of polysaccharides, which lowers the isoelectric point of protein. Moreover, the restricted attack of hydrophilic polysaccharide groups to the protein should inhibit the self‐cross‐linking of proteins (de Oliveira et al., 2016).

### 3.2 Emulsifying stability

The emulsifying stability of rice protein samples from rice dregs was studied under different pH conditions, as shown in Figure 1b. PMR had higher emulsifying stability at pH 2–12 and was, on average, about 10% higher than that of RDP. The emulsifying stability of PMR decreased gradually from pH 2 to pH 6 and increased between pH 6 and pH 12. PMR and RDP produced maximum emulsifying stability of 75.43% and 54.77%, respectively, at pH 12. In other words, the change of emulsifying stability under different pH conditions indicated an improved emulsifying capacity and a positive correlation between the emulsifying stability and solubility of PMR. As previously proposed by Cao, Wen, Li, and Gu (2009), the emulsifying stability of RDPs was influenced by protein solubility and surface hydrophobicity, and PMR showed better emulsifying stability than the blank samples. In fact, the grafting reaction of sodium alginate to RDP probably causes to aggregation of some unfolded protein molecules and inhibits the interaction between unfolded protein molecules. Therefore, PMR has better emulsify effects than the mixture of RDP and sodium alginate (Pirestani, Nasirpour, Keramat, Desobry, & Jasniewski, 2017). In addition, the emulsion stability of PMR was confirmed by the formation of reducing droplet size in terms of the steric effects related to sodium alginate around the oil droplets.

### 3.3 Amino acid analysis

The analysis of amino acids of RDP and PMR is shown in Table 1. It can be discerned a significant decrease in the contents of Lys, Arg, and Cys by 16.62%, 40.07%, and 98.51%, respectively, in PMR as compared to RDP. Meanwhile, the contents of Ser, His, and Tyr decreased by 6.21%, 9.25%, and 5.40%, respectively. This indicated that Lys, Arg, and Cys of RDP played a major role in the Maillard reaction between RDP and sodium alginate under microwave heating, leading to improved solubility and emulsifying capacity (Du et al., 2013). Furthermore, Ser, His, and Tyr should help to improve the protein aggregation. As previously proposed by Li et al. (2013) and Du et al. (2013), the amino groups of RDPs can be reacted with the reducing‐end carbonyl groups of sodium alginate, leading to the glycation reaction between rice dreg and sodium alginate. For example, in a heat treatment process such as microwave heating, the ε‐NH_3_ and the α‐NH_3_ are commonly the major groups during the Maillard reaction (Cao et al., 2009).

**Table 1:** Comparison of amino acid composition of rice dreg, RDP, and PMR

Amino acids	PMR g/100 g protein	RDP g/100 g protein	Rice dreg g/100 g protein
Asp	10.22 ± 1.12^a^	8.76 ± 0.88^b^	10.49 ± 1.01^a^
Glu	22.05 ± 2.51^a^	20.50 ± 2.12^ab^	19.69 ± 1.95^b^
Ser	3.93 ± 0.63^ab^	4.19 ± 0.57^a^	3.87 ± 0.40^b^
His	1.94 ± 0.15^c^	2.14 ± 0.30^b^	2.36 ± 0.32^a^
Gly	5.34 ± 0.75^a^	4.85 ± 0.57^b^	4.43 ± 0.37^c^
Thr	2.25 ± 0.43^c^	3.30 ± 0.42^a^	3.03 ± 0.35^b^
Arg	4.98 ± 0.62^b^	8.31 ± 0.82^ab^	8.72 ± 0.80^a^
Ala	6.09 ± 0.81^a^	5.70 ± 0.77^b^	5.55 ± 0.67^c^
Tyr	4.03 ± 0.54^c^	4.26 ± 0.59^b^	4.52 ± 0.45^a^
Cys	0.01 ± 0.00^b^	0.67 ± 0.06^a^	0.73 ± 0.07^a^
Val	7.07 ± 0.86^a^	6.76 ± 0.83^b^	6.56 ± 0.77^c^
Met	3.33 ± 0.42^a^	3.18 ± 0.38^ab^	2.83 ± 0.17^b^
Phe	6.26 ± 0.77^a^	5.90 ± 0.71^b^	5.42 ± 0.49^c^
Ile	5.22 ± 0.68^a^	4.99 ± 0.60^ab^	4.85 ± 0.53^b^
Leu	8.85 ± 0.91^a^	8.49 ± 0.73^b^	8.15 ± 0.73^c^
Lys	3.11 ± 0.52^b^	3.73 ± 0.61^a^	2.99 ± 0.40^b^
Pro	5.30 ± 0.72^b^	4.27 ± 0.55^c^	5.81 ± 0.61^a^

Note

The different lowercase superscript letters in a row mean significant differences (*p* < 0.05).

### 3.4 Molecular weight

To further study the effect of Millard reaction on the formation of PMR, HPGPC was used to detect molecular weight distribution at 280 nm using UV detector. Chromatographs of PMR, RDP, and rice dreg revealed a significant difference among the molecular weight distributions (Table 2). The high‐molecular‐weight groups increased with the formation of the RDP‐sodium alginate conjugation, but the proportion of low‐molecular‐weight species decreased.

**Table 2:** Range of molecular weight of the rice dreg, RDP, and its grafted product by HPGPC

Relative molecular weight (Da)	Rice dreg (%)	RDP (%)	PMR (%)
>5,000	52.83 ± 1.23^a^	9.55 ± 0.42^b^	54.15 ± 1.28^a^
5,000–3,000	12.67 ± 0.52^a^	11.30 ± 0.47^b^	5.82 ± 0.29^c^
3,000–2,000	6.02 ± 0.31^b^	7.55 ± 0.35^a^	4.32 ± 0.18^c^
2,000–1,000	7.33 ± 0.36^c^	12.49 ± 0.53^a^	8.36 ± 0.37^b^
1,000–500	5.22 ± 0.24^c^	12.34 ± 0.51^a^	8.53 ± 0.37^b^
500–180	4.55 ± 0.21^c^	11.12 ± 0.47^a^	8.91 ± 0.37^b^
<180	11.38 ± 0.49^b^	35.65 ± 0.96^a^	9.90 ± 0.42^c^

Note

The different lowercase superscript letters in a row mean significant differences (*p* < 0.05).

The relative proportions of fractions larger than 5,000 Da between PMR, RDP, and rice dreg were 54.15%, 9.55%, and 52.83%, respectively. Moreover, the proportion of fractions larger than 5,000 Da in PMR increased by 4.53 times as that of RDP. Inversely, the proportion of fractions less than 180 Da decreased markedly from 35.65% to 9.90%. It was probably due to the reaction between the amino groups of the peptides from RDP aggregates and the carbonyl groups of sodium alginate (Pirestani et al., 2017). According to the data in Table 2, the proportions of fractions between 180 and 5,000 Da decreased by 19.87%, 30.88%, 33.07%, 42.78%, and 48.50%, respectively. It is noteworthy that the molecular weight increased with the conjugation between sodium alginate and RDP. On the other hand, there was a positive correlation between the glycosylation and the content of fractions larger than 5,000 Da, and a negative correlation between the glycosylation and the content of fractions less than 5,000 Da. Except for molecular weight distribution, more research is needed to further understand the formation mechanisms of the degradation compounds.

### 3.5 Circular dichroism spectroscopy

CD spectra in far‐UV region (190–250 nm) are often used as an indicator of the changes of proteins secondary structure such as α‐helix, β‐fold, β‐turn, and random coil. Therefore, CD spectroscopy was applied to characterize the spatial conformation of the Maillard reaction products between RDP and sodium alginate. Table 3 shows the secondary structure of PMR. Based on the data of Table 3, the grafting reaction of sodium alginate to RDP had a significant effect on the secondary structure contents of RDP. According to the table, the RDP was composed of 1.42% α‐helix, 15.46% β‐pleated sheet, 17.16% β‐turn, and 65.58% random coil, confirming that the major secondary structures in RDP were β‐pleated sheet, random coil, and β‐turn. For PMR, its secondary structure was different from those of RDP, further revealing the reaction between RDP and sodium alginate under microwave heating. However, a significant difference was observed in the secondary structure of PMR. It had a lower proportion of β‐pleated sheets (from 15.63% to 4.54%) and β‐turns (from 17.16% to 2.37%), but a significant increase was observed in α‐helices (from 1.42% to 4.24%) and random coils (from 65.58% to 88.95%), indicating that random coils accounted for the dominated secondary structure of PMR. The underlying cause may be that the introduction of more hydroxyl groups from sodium alginate to RDP resulted in the development of hydrogen bonds between RDP and sodium alginate molecules, which could inhibit neighboring proteins from the interaction with each other and therefore led to an increase in α‐helix and a decrease in β‐turn and β‐pleated sheet (Du et al., 2013). Meanwhile, it was proposed that the conjugation of RDP with sodium alginate may lead to unfolding and conformational flexibility and changed spatial structure of proteins, which may alter their functional properties.

**Table 3:** Secondary structure of RDP, RDP, and PMR

	α‐helix (%)	β‐pleated sheet (%)	β‐turn (%)	Random coil (%)
Rice dreg	5.41 ± 0.25^a^	47.98 ± 0.78^a^	29.40 ± 0.56^a^	24.09 ± 0.52^c^
RDP	1.42 ± 0.11^c^	15.63 ± 0.43^b^	17.16 ± 0.45^b^	65.58 ± 0.81^b^
PMR	4.24 ± 0.20^b^	4.54 ± 0.22^c^	2.37 ± 0.17^c^	88.95 ± 0.86^a^

Note

The different lowercase superscript letters in a column mean significant differences (*p* < 0.05).

### 3.6 FTIR spectra

**Figure 2:**
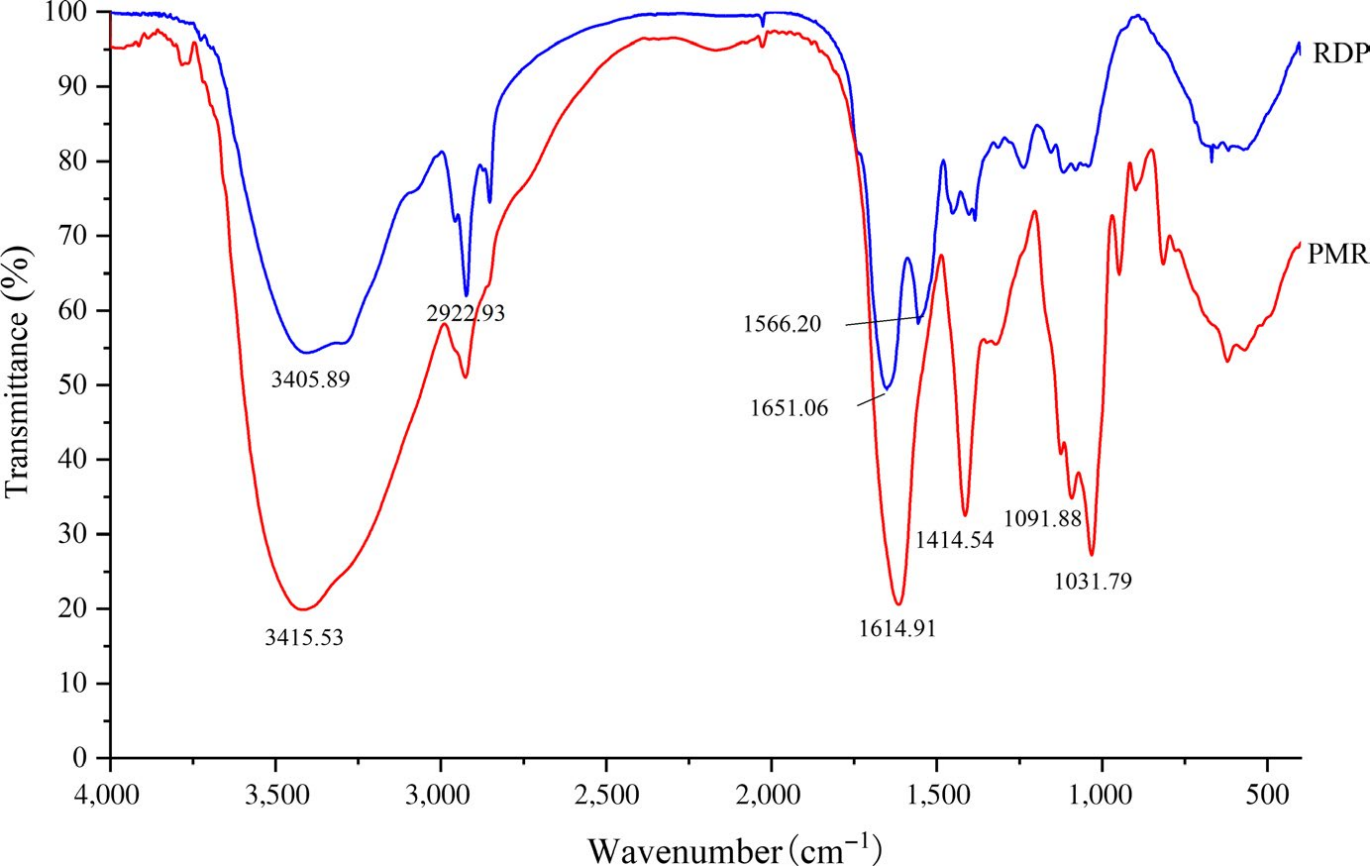
FTIR spectra of RDP and PMR

The FTIR spectra of PMR and RDP are shown in Figure 2. When RDP is grafted with sodium alginate by a covalent bond, there were several distinguishable changes in the FTIR spectrum ascribing to the consumption of some functional groups, such as a significant increase in hydroxyl content in the conjugates. There are two distinctive spectral features for hydroxyl groups, namely the stretching vibration of O–H at 3,700–3,200 cm^−1^ and the stretching vibration of C–O at 1,100–1,000 cm^−1^. As shown in Figure 2, upon modification by the Maillard reaction, strong absorption was, respectively, observed at 3,415, 1,091, and 1,031 cm^−1^. Meanwhile, the absorption band of PMR at 3,415 cm^−1^ due to stretching vibration of O–H was more intense than that of RDP, indicating that the amount of hydroxyl groups and hydrogen bonding in PMR greatly increased as grafting reaction progress, which was owing to the increased sodium alginate grafted to RDP molecules (Li et al., 2013; Yao et al., 2018). Meanwhile, the new strong absorption was observed at 1,031 cm^−1^, indicating a bending vibration of O–H and a strong stretching vibration of new C–N occurred in conjugates, which may be ascribed to the combination of sodium alginate with RDP. The characteristic absorption peaks of protein include bending vibration of N–H at 1,680–1,630 cm^−1^, bending vibration of C–NH at 1,530–1,560 cm^−1^, and stretching vibration of C–N and bending vibration of N–H at 1,240–1,450 cm^−1^. The characteristic absorption peaks appeared at 1,651 and 1,556 cm^−1^ for RDP. On the other hand, the grafted product did not show absorption peaks at either site, but there was a strong C–N stretching at 1,414 cm^−1^, indicating the NH_2_‐group of RDP covalently bonded with sodium alginate, resulting in a decrease of NH_2_‐group content in the protein molecule. In addition, spectral changes at 800–500 cm^−1^ could also demonstrate the alteration of protein structures. All of the above analysis suggests that the conjugation of RDP with sodium alginate had obvious effects on the molecular structure of RDP.

### 3.7 Immune activities of PMR

**Figure 3:**
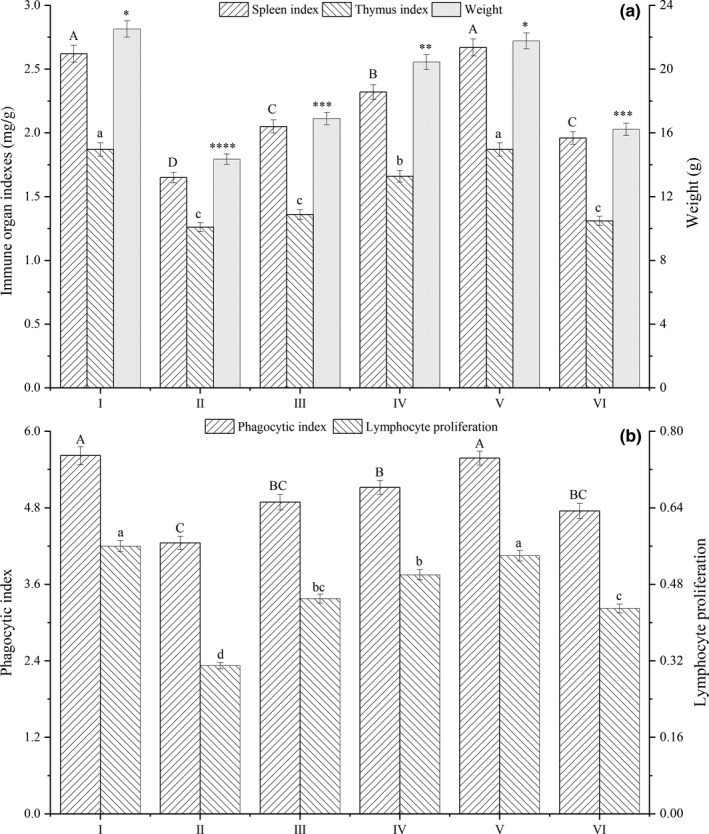
Effect of PMR on spleen index, thymus index, phagocytic index, and lymphocyte proliferation of immunodeficient mice. I: Blank; II: Model group; III: PMR + CY group (80 mg/kg); IV: PMR + CY group (160 mg/kg); V: PMR + CY group (320 mg/kg). ^A–D, a–d^The different superscript letters in bars mean significant differences between different treatment groups (*p* < 0.05)

The thymus plays a vital role in the immune regulation on immunodeficient mice, maintaining T‐cell differentiation and maturation. The spleen is regarded as the largest immune organ, promoting hematopoiesis. And the spleen could facilitate the development and maturation of T cells and B cells through the immune response when stimulated by antigens. In addition, the spleen could promote the production and secretion of certain bioactive factors, such as interferon, cytokines, and complement. Thus, the body's immune function could be indirectly reflected by immune organ indexes. In this study, the effect of PMR on immune function of immunodeficient mice was shown in Figure 3. As shown in Figure 3a, compared to the blank group (untreated with CY), the thymus index and spleen index of immunodeficient mice (model group) decreased significantly (*p* < 0.05) by 37.02% and 32.62%, respectively. However, the thymus index and spleen index of immunodeficient mice could be improved by PMR. For example, the thymus index and spleen index of immunodeficient mice treated with a constant daily dose of 80 mg PMR per kg body weight were 2.05 and 1.36, which were 24.24% and 7.94% higher, respectively, than those of the model group. Meanwhile, there was a concentration dependent in the immune activities of PMR, generally enhanced with the increase of concentrations. The results revealed that PMR could promote the growth of immune organs of immunodeficient mice, leading to the improvement of immunity functions.

Lymphocyte proliferation which is the essential stage of the immune response could be used to directly reflect the cellular immunity function. It could induce cell to produce stimulated lymphocytes and eliminate alloantigen. Usually, lymphocyte proliferation is used to evaluate the immune regulation activity of active substances. As shown in Figure 3b, in comparison with the blank group (untreated with CY), the phagocytic index and lymphocyte proliferation decreased significantly by 24.38% and 44.64%, respectively, in immunodeficient mice (model group). However, the phagocytic index and lymphocyte proliferation of immunodeficient mice could be noticeably improved by PMR treatment. For example, the phagocytic index and lymphocyte proliferation of immunodeficient mice treated with a constant daily dose of 80 mg PMR per kg body weight were 4.89 and 0.45, respectively, which were 24.24% and 7.94% higher, respectively, than that of the model mice. Meanwhile, there was a concentration dependent in the immune regulation of phagocytic index and lymphocyte proliferation, generally enhanced with the increase of concentrations of PMR.

In summary, based on the immunodeficient mice experiments, it was found that PMR could play a vital role in the immune regulation of immunodeficient mice. The immunomodulation of PMR was concentration dependent and generally enhanced with the increase of concentrations of PMR. This suggests that glycosylation modification of RDP by wet heating assisted with microwave treatment can not only improve the performance of the final product and expand the application field of RDP, but also bring certain bioactivity benefits. Further investigation of the glycosylation products of RDP to characterize the effective structure–function relationships and mechanisms might lead to the development of nutraceuticals indicated for promoting immunomodulation.

## 4 CONCLUSION

The glycosylation modification of RDP with sodium alginate by wet heating assisted with microwave treatment is an effective method. The solubility of PMR was significantly increased by grafting RDP with sodium alginate. Meanwhile, under the same grafted conditions, the emulsifying capacity of PMR was also improved, when compared to that of RDP. Based on structural analysis of PMR, it was concluded that the amino groups of RDP can be reacted with the reducing‐end carbonyl groups of sodium alginate, leading to unfolding and conformational flexibility of RDP and spatial structure changes and consequently improved solubility and emulsifying capacity of RDP grafted with sodium alginate. Furthermore, it can be concluded from the results that RDP grafted with sodium alginate had strong immunomodulatory properties. Therefore, the glycosylation modification of protein with polysaccharide is a promising and effective way to develop novel techno‐functional additives in a wide range of food industry, cosmetic, and pharmaceutical formulations as added‐value food ingredients.

## CONFLICT OF INTEREST

All authors declare no conflict of interest.

## ETHICAL STATEMENTS

All animals were housed and cared for in accordance with the Chinese Pharmacological Society Guidelines for Animal Use.
